# Disrupted Glucose Metabolism Covariance Network in Amyotrophic Lateral Sclerosis

**DOI:** 10.1111/cns.70537

**Published:** 2025-07-28

**Authors:** Xin Jin, Xueying Wang, Dingxin Zheng, Pubing Yuan, Jianyu Li, Ting Qiu, Huixiong Zhang, Yifan Chen, Jinfan Zhang, Feifei Wu, Qing Liu, Alessandro Grecucci, Yuanchao Zhang, Junling Wang, Xiaoping Yi, Lena Palaniyappan, B. Blair Braden

**Affiliations:** ^1^ The Clinical Hospital of Chengdu Brain Science Institute, MOE Key Lab for Neuroinformation University of Electronic Science and Technology of China Chengdu Sichuan P. R. China; ^2^ School of Life Science and Technology University of Electronic Science and Technology of China Chengdu Sichuan P. R. China; ^3^ Department of Radiology Xiangya Hospital, Central South University Changsha Hunan P. R. China; ^4^ Douglas Mental Health University Institute McGill University Montreal Quebec Canada; ^5^ Department of Neurology Xiangya Hospital, Central South University, Jiangxi (National Regional Center for Neurological Diseases) Nanchang Jiangxi P. R. China; ^6^ Department of Psychology and Cognitive Sciences (DiPSCo) University of Trento Rovereto Italy; ^7^ College of Health Solutions Arizona State University Phoenix Arizona USA; ^8^ Hunan International Scientific and Technological Cooperation Base of Neurodegenerative and Neurogenetic Diseases Changsha Hunan P. R. China; ^9^ National Clinical Research Center for Geriatric Diseases, Xiangya Hospital Central South University Changsha Hunan P. R. China; ^10^ Key Laboratory of Hunan Province in Neurodegenerative Disorders Central South University Changsha Hunan P. R. China; ^11^ Center for Medical Genetics, School of Life Sciences Central South University Changsha Hunan P. R. China; ^12^ Engineering Research Center of Hunan Province in Cognitive Impairment Disorders Central South University Changsha Hunan P. R. China; ^13^ Department of Radiology Chongqing University Three Gorges Hospital, Chongqing University Chongqing P. R. China; ^14^ Clinical Research Center (CRC), Medical Pathology Center (MPC), Cancer Early Detection and Treatment Center (CEDTC) and Translational Medicine Research Center (TMRC) Chongqing University Three Gorges Hospital, Chongqing University Chongqing P. R. China; ^15^ School of Medicine Chongqing University Chongqing P. R. China; ^16^ FuRong Laboratory Changsha Hunan P. R. China

**Keywords:** ALS, excitotoxity, glutamate, graph‐theoretic analysis, TDP‐43

## Abstract

**Aims:**

This study aimed to characterize the topological changes in glucose metabolism covariance networks in amyotrophic lateral sclerosis (ALS).

**Methods:**

We assessed the interregional coordination of ^18^F‐FDG‐PET data to examine topological alterations in individualized glucose metabolism covariance networks in 127 ALS patients compared to 128 healthy controls (HC).

**Results:**

Compared to HC, ALS patients showed reduced small‐worldness (lower normalized clustering coefficient, higher normalized characteristic path length) and decreased global and local efficiency, suggesting impaired global integration and local segregation. These network metrics correlated with disease progression and motor function. Regionally, altered degree centrality affected motor and default mode networks, and related to GABAa and mGluR5 receptor expression. Transcriptomic associations further linked these changes to immune function, synaptic signaling, and protein regulation. Bidirectional shifts in connectivity strength were observed, with both increased connectivity and disease progression independently predicting reduced survival.

**Conclusion:**

Our findings may provide valuable biomarkers for monitoring the progression of ALS and suggest potential mechanistic pathways for the development of innovative therapeutic strategies for this disorder.

## Graphical abstract

This study reveals that metabolism covariance networks in ALS patients exhibit a suboptimal small‐world architecture, with widespread nodal and connectional changes. These changes are associated with disease progression and are linked to receptor expression and immune processes. The results provide potential biomarkers for monitoring ALS progression.

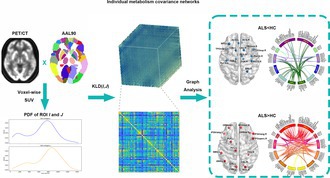

## 1 Introduction

Amyotrophic lateral sclerosis (ALS) is a devastating neurodegenerative disorder that affects both upper and lower motor neurons, resulting in limb paralysis, dysarthria, dysphagia, and death within 3–5 years due to respiratory failure [1, 2]. Besides, ALS is often accompanied by variable extramotor neurodegeneration, compromising behavior, language, and cognitive functions. Indeed, around 50% of cases suffer from cognitive and behavioral changes, with up to 14% meeting the criteria for comorbid frontotemporal dementia (FTD) [3, 4].

Neuroimaging investigations have demonstrated prominent structural and functional brain abnormalities that underlie the motor and nonmotor manifestations in ALS. For example, structural MRI studies have revealed decreased gray matter volume and thickness in the precentral gyrus [5], orbitofrontal [6], and temporal cortices [7]. Functional MRI studies have identified attenuated functional connectivity in sensorimotor circuits and in networks associated with behavior and cognition [8]. Positron emission tomography (PET) studies have uncovered a mixed pattern of metabolic changes, showing hypometabolism in the primary motor cortex and frontotemporal lobe, hypermetabolism in the paracentral lobe, and a coexistence of hypometabolism and hypermetabolism in the occipital lobe [9, 10, 11]. These findings suggest that ALS is not merely a disorder confined to the motor system but rather a multisystem disease impacting large‐scale brain networks.

Structural and functional covariance networks constructed using morphological or functional parameters provide a powerful framework for characterizing topological anomalies of large‐scale networks associated with psychiatric and neurodegenerative diseases [12, 13, 14, 15]. These networks depict intra‐ and inter‐individual covariation in distinct brain areas shaped by neurodevelopmental factors such as genetics and environment. Through assessing interregional covariance of gray matter volumes, our previous structural covariance network analysis uncovered weakened global integration yet enhanced local segregation in ALS, suggesting a coexistence of both neurodegenerative and compensatory mechanisms. However, it was limited by group‐level network construction, preventing identification of clinical correlates of network alterations. Furthermore, abnormalities in structural covariance networks are observable only in patients at more advanced stages, potentially limiting the effectiveness of interventions in reversing these alterations. In contrast, functional covariance networks exhibit greater sensitivity in detecting instantaneous abnormalities before specific brain regions undergo irreversible structural changes. To our knowledge, no prior study has conducted functional covariance network analyses in ALS. Investigating changes in individualized covariance networks using sensitive functional parameters such as glucose uptake, as well as their neurotransmitter correlates and transcriptional bases may shed light on the pathophysiological mechanisms of ALS and contribute potential pathways for developing novel intervention strategies aimed to slow disease progression at an early stage.

This study aimed to characterize abnormalities in glucose metabolism covariance network in a large cohort of ALS patients compared to healthy controls (HC). Specifically, an individualized glucose metabolism covariance network was constructed for each participant by assessing the distributional similarity of voxel uptake values from ^18^F‐FDG‐PET images for every two regions on the automated anatomical labelling (AAL) atlas [16]. Graph theoretical and functional connectivity analyses were conducted for glucose metabolism covariance networks between the two groups. We hypothesized that the glucose metabolism covariance network of ALS patients would exhibit a suboptimal topological organization as evidenced by changes in global (such as decreases in efficiency parameters) and nodal (degree centrality) network parameters. We also hypothesized that ALS patients would show bidirectional changes in metabolic connectivity, suggesting a coexistence of neurodegeneration and compensatory mechanisms.

## 2 Materials and Methods

### 2.1 Participants

One hundred twenty‐seven ALS patients were recruited from the Department of Neurology at Xiangya Hospital, Central South University, China. The inclusion criteria required a diagnosis of clinically definite, probable, or probable laboratory‐supported ALS, confirmed by two experienced neurologists following the revised 2015 El Escorial criteria [17]. Only patients able to complete a brain PET/CT scan and without comorbid neurological or psychiatric conditions were included. Demographic and clinical data were collected at the time of screening, including age at onset, site of onset, time from onset to diagnosis, and scores on the Amyotrophic Lateral Sclerosis Functional Rating Scale‐Revised (ALSFRS‐R), Mini‐Mental State Examination (MMSE), Generalized Anxiety Disorder 7‐item (GAD‐7) scale, and Patient Health Questionnaire‐9 (PHQ‐9). Progression rate (ΔFS) was calculated using the formula: (48‐ALSFRS‐R score at baseline)/disease duration from onset of symptoms (months). Overall survival was calculated from the time of diagnosis to either the time of death or the last follow‐up at the end of the study. For comparison, 128 matched HC were included, defined as individuals who: (i) had no history of oncologic, neurological, or psychiatric illness; (ii) had normal brain 18F‐FDG‐PET scans confirmed by two experienced nuclear medicine physicians; and (iii) showed no neurological abnormalities on examination. Demographic and clinical details are in Table 1.

**TABLE 1:** Demographic and clinical features of ALS patients and HC.

	ALS patients (*n* = 127)	HC (*n* = 128)	Statistic	*p*
Age, years	55.65 ± 9.94	55.24 ± 7.75	*t* = 0.36	0.72
Male/Female	87/40	88/40	χ^2^ = 0	1
Age at onset, years	54.70 ± 10.04			
Onset site (Limb/Bulbar/Other)	90/30/7			
Diagnostic delay, months	12.94 ± 11.72			
OS, months	27.55 ± 18.05			
ALSFRS‐R	39.32 ± 5.72			
ΔFS	1.07 ± 0.94			
MMSE	22.37 ± 6.18	25.97 ± 1.84	*t* = 6.31	< 0.001
PHQ‐9	8.16 ± 8.30	4.10 ± 1.47	*t* = 5.46	< 0.001
GAD‐7	7.42 ± 6.44	4.52 ± 1.98	*t* = 4.88	< 0.001

*Note:* Data represent mean ± standard deviation. Between‐group differences were examined using two‐sample *t*‐test and χ^2^ test. Limb onset refers to single or bilateral involvement of the upper or lower limbs (e.g., arm, leg, or combinations). Bulbar onset refers to cases with brainstem‐related symptoms (e.g., dysarthria, dysphagia). Other onset includes atypical sites such as the trunk, facial sensory symptoms, or respiratory involvement.

Abbreviations: ALSFRS‐R, Amyotrophic Lateral Sclerosis Functional Rating Scale‐Revised; GAD‐7, General Anxiety Disorder‐7; MMSE, Minimum Mental State Examination; OS, overall survival; PHQ‐9, Patient Health Questionnaire‐9; ΔFS, progression rate of ALS.

### 2.2 ^18^F‐FDG‐PET Acquisition and Preprocessing

Brain ^18^F‐FDG‐PET scans were performed using a Discovery Elite PET/CT scanner following standard protocols. Participants fasted for at least 6 h before the scan, ensuring fasting blood glucose levels below 7.2 mmol/L. A dose of 3.7 MBq/kg of ^18^F‐FDG was administered intravenously through the cubital vein over 1 min. Scanning commenced 60 min post‐injection, with three‐dimensional PET images acquired over 5 min.

Participants were positioned so that image slices aligned parallel to the canthomeatal line. Images were reconstructed into a 256 × 256 trans‐axial matrix using the 3D VUE Point ordered‐subset expectation–maximization algorithm (6 iterations, 6 subsets), producing 47 trans‐axial slices at 3.25‐mm intervals. A simultaneous low‐dose CT scan was acquired for attenuation correction. Participants remained under observation for 90 min post‐injection and were instructed to report any adverse effects.

Image processing was conducted using SPM12 (https://www.fil.ion.ucl.ac.uk/spm/software/spm12). Individual PET volumes were normalized to the Montreal Neurological Institute space and resampled to 1 × 1 × 1 mm^3^. Standardized uptake values were utilized to construct individual metabolic covariance networks.

### 2.3 Glucose Metabolism Covariance Networks Construction

Ninety regions of interest (ROIs) from AAL atlas were considered, excluding the cerebellum, to construct individual glucose metabolism covariance networks (Table S1). For each ROI, we estimated the probability distribution function (PDF) of the standardized uptake values by calculating histograms of voxel intensities within that region. We then evaluated the symmetric Kullback–Leibler divergence (KLD) of the PDFs for each pair of ROIs using the following formula (1), where *P* and *Q* represent the PDFs of the two corresponding ROIs.

(1) $$D_{\mathrm{KL}}\left(P,Q\right)=\frac{1}{2}\sum_{i}\left(P\left(i\right)\log\frac{P\left(i\right)}{Q\left(i\right)}+Q\left(i\right)\log\frac{P\left(i\right)}{Q\left(i\right)}\right)$$

For intergroup comparison, the $D_{\mathrm{KL}}\left(P,Q\right)$ value was divided by the mean KLD across all ROI pairs to obtain a normalized symmetric KLD, denoted as $\bar{D_{\mathrm{KL}}\left(P,Q\right)}$. This normalized divergence quantifies the difference between the PDFs of two ROIs. To convert this metric into a similarity measure constrained within a range of 0 and 1, the normalized KLD underwent an exponential transformation, represented by the following equation (2).

(2) $$S_{\mathrm{KL}}\left(P,Q\right)=e^{-\bar{D_{\mathrm{KL}}\left(P,Q\right)}}$$

This transformation yields a 90 × 90 adjacency matrix for each participant, where the element in the *i*‐th row and *j*‐th column reflects the metabolic connection strength between ROI‐*i* and ROI‐*j*.

### 2.4 Network Properties Analysis

We assessed the topological organization of glucose metabolism covariance networks using five global parameters—small‐world index (Sigma), normalized clustering coefficient (Gamma), normalized characteristic path length (Lambda), global efficiency (Eg), and local efficiency (Eloc)—representing small‐worldness, resilience, segregation, and integration. Degree centrality (DC) was calculated as a nodal metric of regional importance. Networks were constructed using a sparsity‐based thresholding approach (range:0.1–0.3; step = 0.01), retaining the strongest S% of edges to ensure equal edge density across participants. Network measures were computed at each sparsity level, and group differences were tested using two‐sample t‐tests on the area under the curve (AUC) values. DC comparisons were corrected for multiple comparisons using the false discovery rate (FDR).

### 2.5 Correlation With Neurotransmitters

To explore whether between‐group deviances in DC relate to an imbalance of excitatory and inhibitory neurotransmitters, we assessed the spatial correlations of the regional *t*‐values from the between‐group contrast of DC with existing nuclear imaging‐derived maps of glutamate (mGluR5) and gamma‐aminobutyric acid (GABAa). Specifically, Pearson's correlation coefficients between the t‐map and the neurotransmitter maps were computed across the 90 regions of the AAL atlas, adjusting for spatial autocorrelation using the “neuromaps” toolbox. The exact *p*‐values were determined using spatial permutation‐based null maps generated from 1000 permutations and were subsequently corrected for multiple comparisons using the Bonferroni method.

### 2.6 Neuroimaging‐Transcriptional Association Analysis

We performed a neuroimaging‐transcriptional association analysis to examine the potential biological processes underlying the intergroup DC deviances. First, we extracted the t‐value for each of the 90 AAL regions from the between‐group contrast of DC, generating a 90 × 1 vector of regional DC deviances. Next, we obtained gene expression data from the Allen Human Brain Atlas and mapped it onto the 90 regions of the AAL atlas, creating a 90 × 16,533 regional transcription matrix. We then performed a partial least squares (PLS) regression to explore the relationship between regional transcription data (predictors) and the DC deviance vector (response). The first component (PLS1) represents the linear combination of gene expression weights that best predicts the neuroimaging feature across regions. To assess the statistical significance of PLS1, a permutation test with 1000 replications was conducted. Bootstrapping (1000 resampling with replacement) was used to estimate the error of each gene's PLS1 weight. Subsequently, we calculated a Z‐score for each gene as the ratio of its weight to its bootstrap standard deviation. Genes with a Z‐score > 3 and those with a Z‐score < −3, indicating lower and higher DC in ALS patients, were separately entered into enrichment analysis using Metascape to identify enrichments in gene ontology (GO) biological processes and Kyoto Encyclopedia of Genes and Genomes (KEGG) pathways.

### 2.7 Edge Connectivity Strength Analysis

Differences in edge connectivity strength were analyzed using a two‐sample *t*‐test. The results were adjusted for multiple comparisons using the Bonferroni correction method.

### 2.8 Cox Proportional Hazards Model Analysis

We fitted a Cox proportional hazards model to evaluate the association between overall survival and various factors. The model incorporated demographic (age, gender), clinical (ALSFRS‐R, ΔFS), and network variables (Gamma, Lambda, Eg, Eloc, average increase/decrease in DC, and average increase/decrease in edge connectivity strength).

## 3 Results

### 3.1 Alterations in Global Network Parameters

**FIGURE 1:**
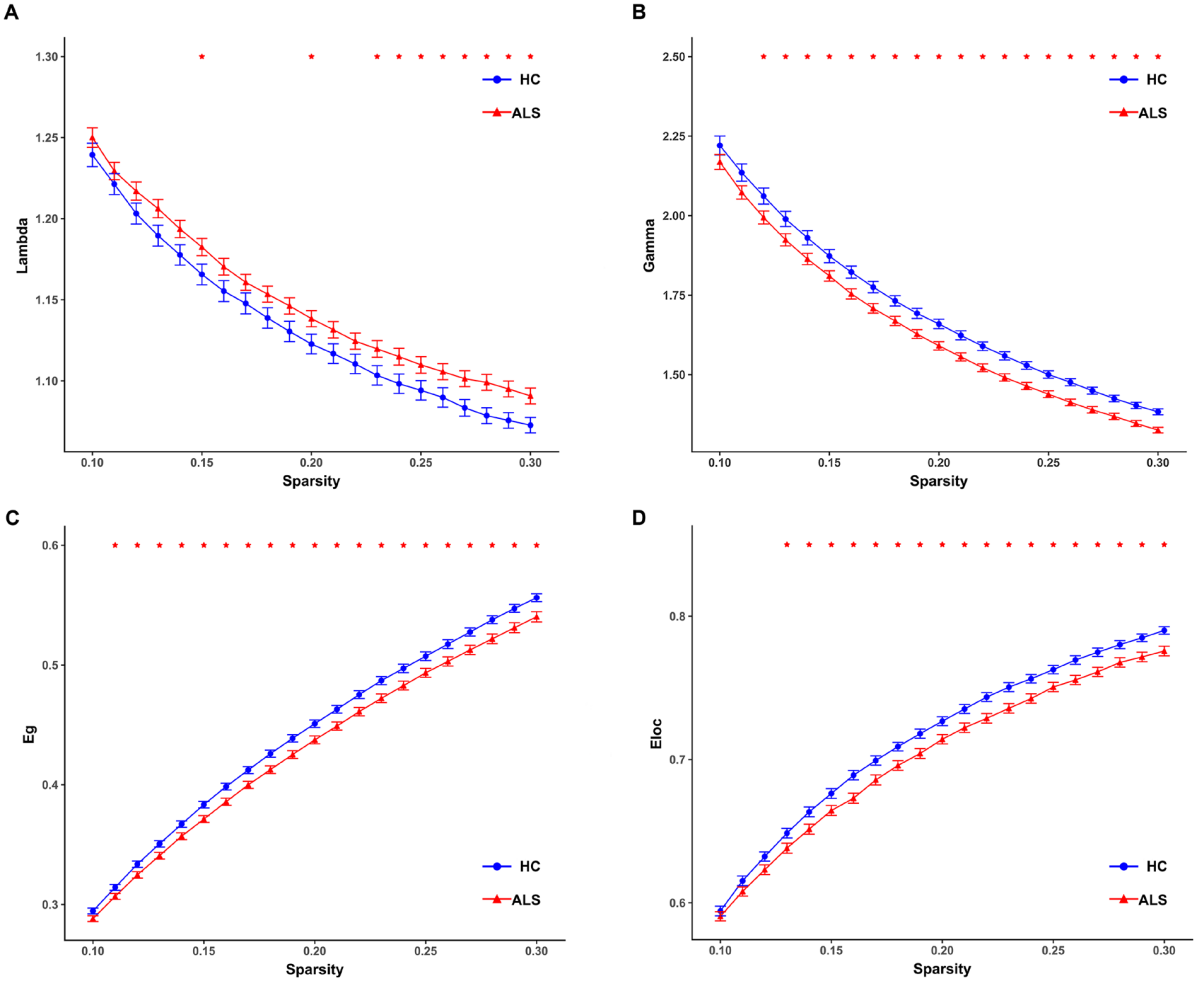
Differences in small‐world indices and network efficiency parameters between ALS patients and HC. ALS patients showed significantly (A) higher normalized characteristic path length (Lambda), as well as (B) lower normalized clustering coefficient (Gamma), (C) global efficiency (Eg), and (D) local efficiency (Eloc) across several sparsity levels.

Both ALS patients and HC exhibited a small‐world topology in their metabolism covariance networks (i.e., Sigma > 1.2; Figure S1). Compared to HC, ALS patients showed increased Lambda and decreased Gamma, Eg, and Eloc at several network sparsity levels (Figure 1). AUC analyses further confirmed the significant decreases in Sigma (*p* < 0.001), Gamma (*p* = 0.002), Eg (*p* = 0.003), and Eloc (*p* = 0.002), along with a significant increase in Lambda (*p* = 0.033) in ALS patients compared to HC.

### 3.2 Alterations in Nodal Network Parameter

**FIGURE 2:**
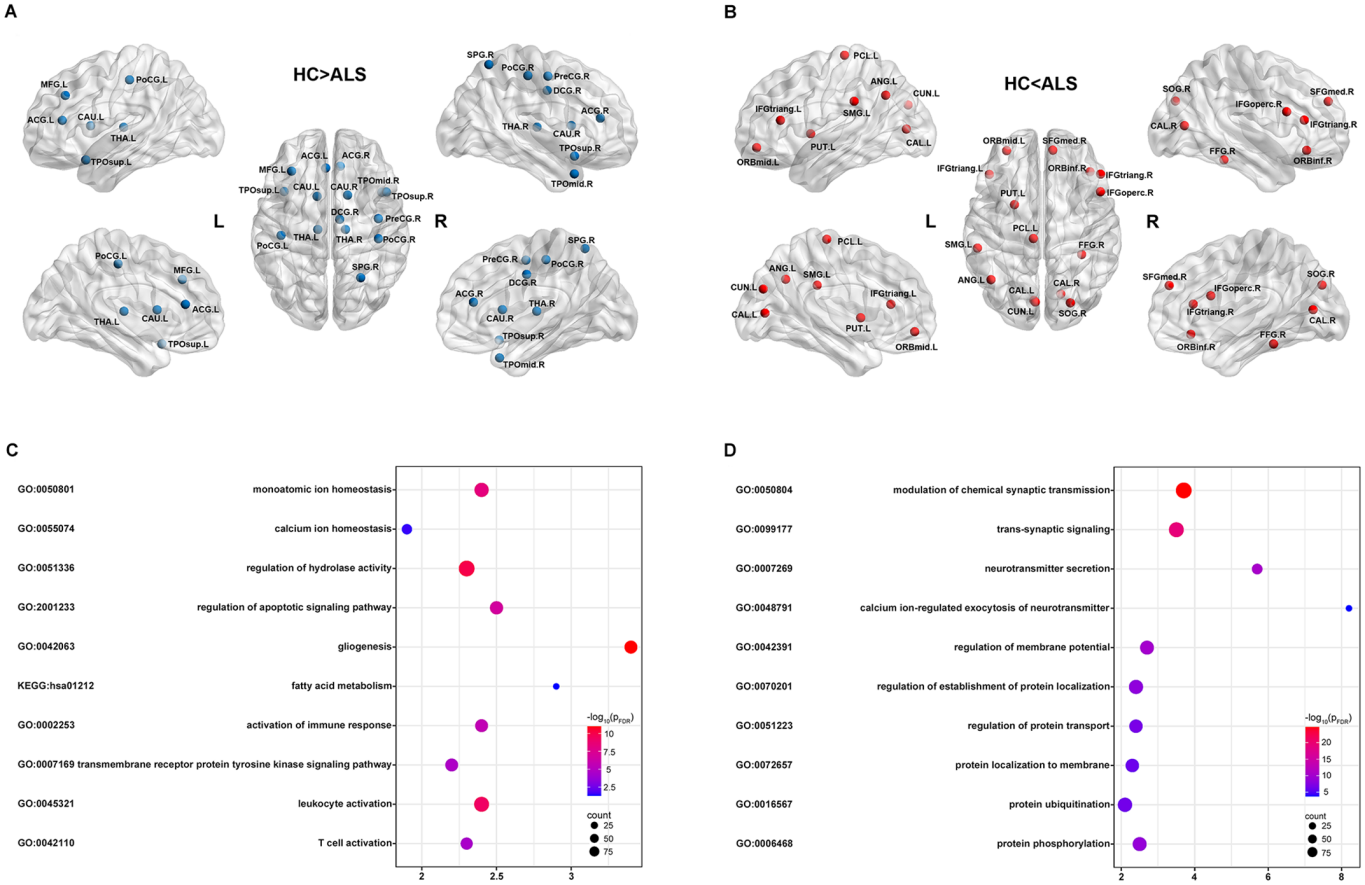
Brain regions showing significant difference in DC between ALS patients and HC and their transcriptional bases. ALS patients showed both (A) decreased and (B) increased DC in multiple brain regions compared to HC. Functional enrichment analyses of the pivotal genes associated with (C) lower and (D) higher DC in ALS patients. Terms were retained with a threshold of false discovery rate (FDR) corrected *p* < 0.05. DC, degree centrality; L, left; R, right.

Compared to HC, we found bidirectional changes in DC in ALS patients (Figure 2; Table S2). Specifically, patients showed decreased DC in the right precentral gyrus; bilateral middle frontal gyrus, postcentral gyrus, anterior and middle cingulate gyrus, thalamus, caudate, superior temporal pole; and right middle temporal pole, while showing increased DC in the left putamen, right pars opercularis, and bilateral pars triangularis, left angular gyrus, right medial superior frontal gyrus, right superior occipital gyrus, among others.

### 3.3 Correlation With Neurotransmitters

Cross‐region spatial correlation analyses demonstrated that the regional *t*‐values from the between‐group contrast of DC were negatively correlated with the expression of the GABAa receptor (*r* = −0.424, *p* = 0.010, Bonferroni‐corrected) and mGluR5 receptor (*r* = −0.126, *p* = 0.012, Bonferroni‐corrected).

### 3.4 Neuroimaging‐Transcriptional Association Analysis

With a threshold of Z‐score > 3, we identified 1662 pivotal genes associated with lower DC in ALS patients. Using this gene list, functional enrichment analysis revealed several biological processes/pathways highly relevant to immune responses (such as “monoatomic ion homeostasis”, “calcium ion homeostasis”, “regulation of hydrolase activity”, “gliogenesis”, “leukocyte activation”, “T cell activation”, “fatty acid metabolism”, “activation of immune response”) (Figure 2C).

With a threshold of Z‐score < −3, we identified 1682 pivotal genes associated with higher DC in ALS patients. These genes were enriched in biological processes/pathways highly relevant to trans‐synaptic signal transduction (such as “trans‐synaptic signaling”, “neurotransmitter secretion”, “synaptic vesicle exocytosis”) and regulation of protein function (such as “regulation of establishment of protein localization”, “protein ubiquitination” and “protein phosphorylation”) (Figure 2D).

### 3.5 Alterations in Edge Connectivity Strength

**FIGURE 3:**
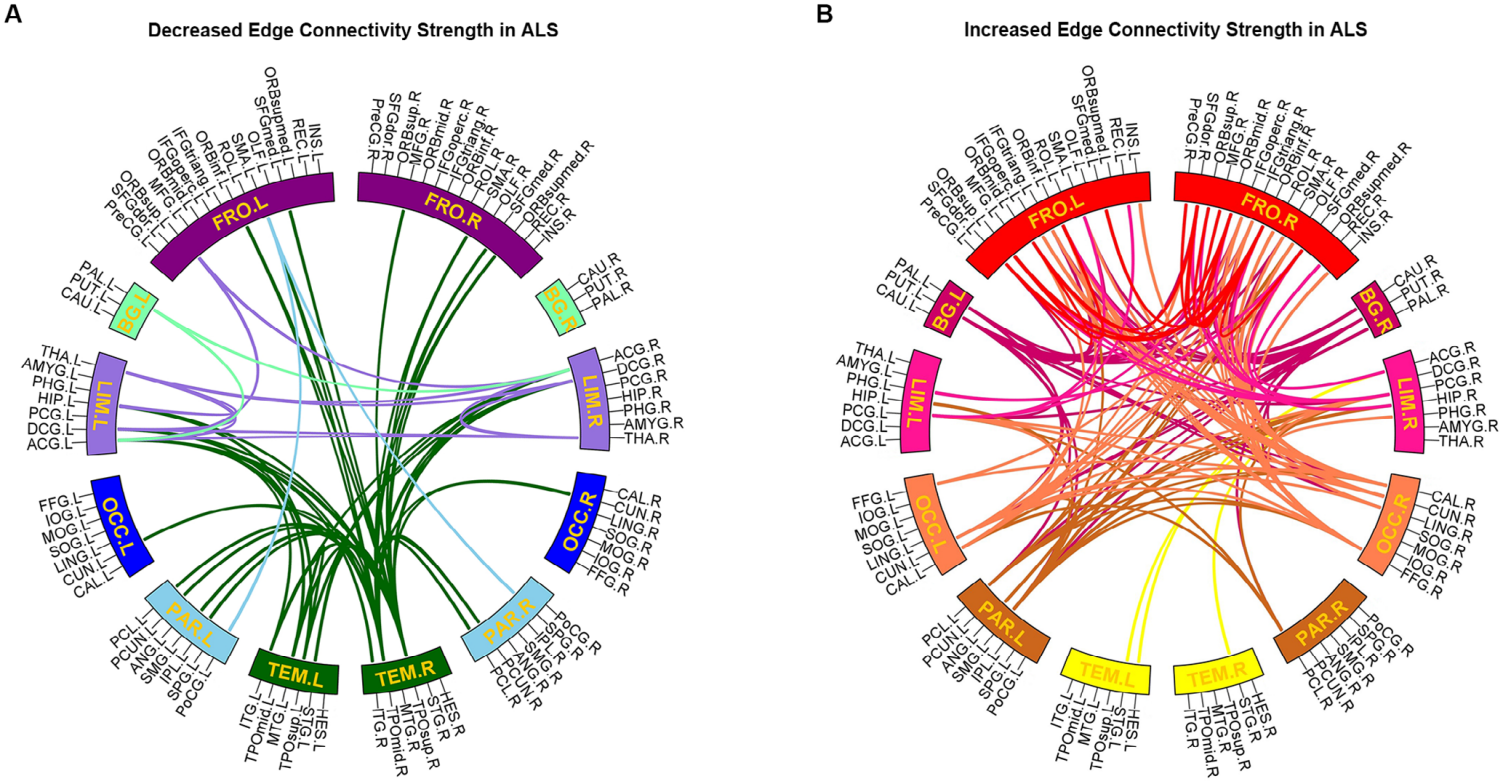
Differences in edge connectivity strength between ALS patients and HC. ALS patients showed both (A) decreased and (B) increased connectivity strength compared to HC. The results were corrected for multiple comparisons using the Bonferroni method.

Compared to HC, ALS patients exhibited both increased and decreased edge connectivity strength (Figure 3; Figure S2; Table S3). Decreased edge connectivity strength was primarily observed between the temporal and frontal lobes, between the temporal lobe and limbic system, between the temporal and parietal lobes, and between the bilateral limbic system. Increased edge connectivity strength was predominantly found between the frontal and occipital lobes, basal ganglia, and limbic system, as well as within the frontal and occipital lobes.

### 3.6 Relationships Between Network Parameters and Clinical Data

**FIGURE 4:**
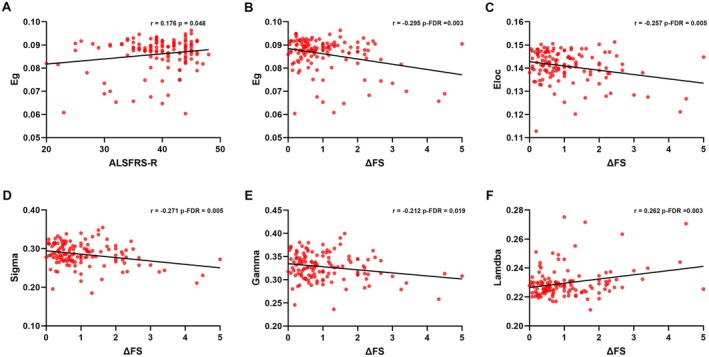
Associations between the AUC of global network parameters and clinical data in ALS patients. Eg, global efficiency; Eloc, local efficiency; Gamma, normalized clustering coefficient; Lambda, normalized characteristic path length; Sigma, small‐world index.

In ALS patients, higher Eg values were associated with greater ALSFRS‐R scores (Figure 4). Additionally, lower values of Eg, Eloc, Sigma, and Gamma were associated with higher ΔFS, while higher Lambda was associated with greater ΔFS.

For nodal network parameter, higher DC values in the right superior occipital gyrus were associated with higher MMSE scores (*r* = 0.322, FDR‐corrected *p* = 0.020), whereas lower DC values in the left postcentral gyrus were associated with greater ΔFS (*r* = −0.263, FDR‐corrected *p* = 0.0286).

### 3.7 Cox Proportional Hazards Model Analysis

**FIGURE 5:**
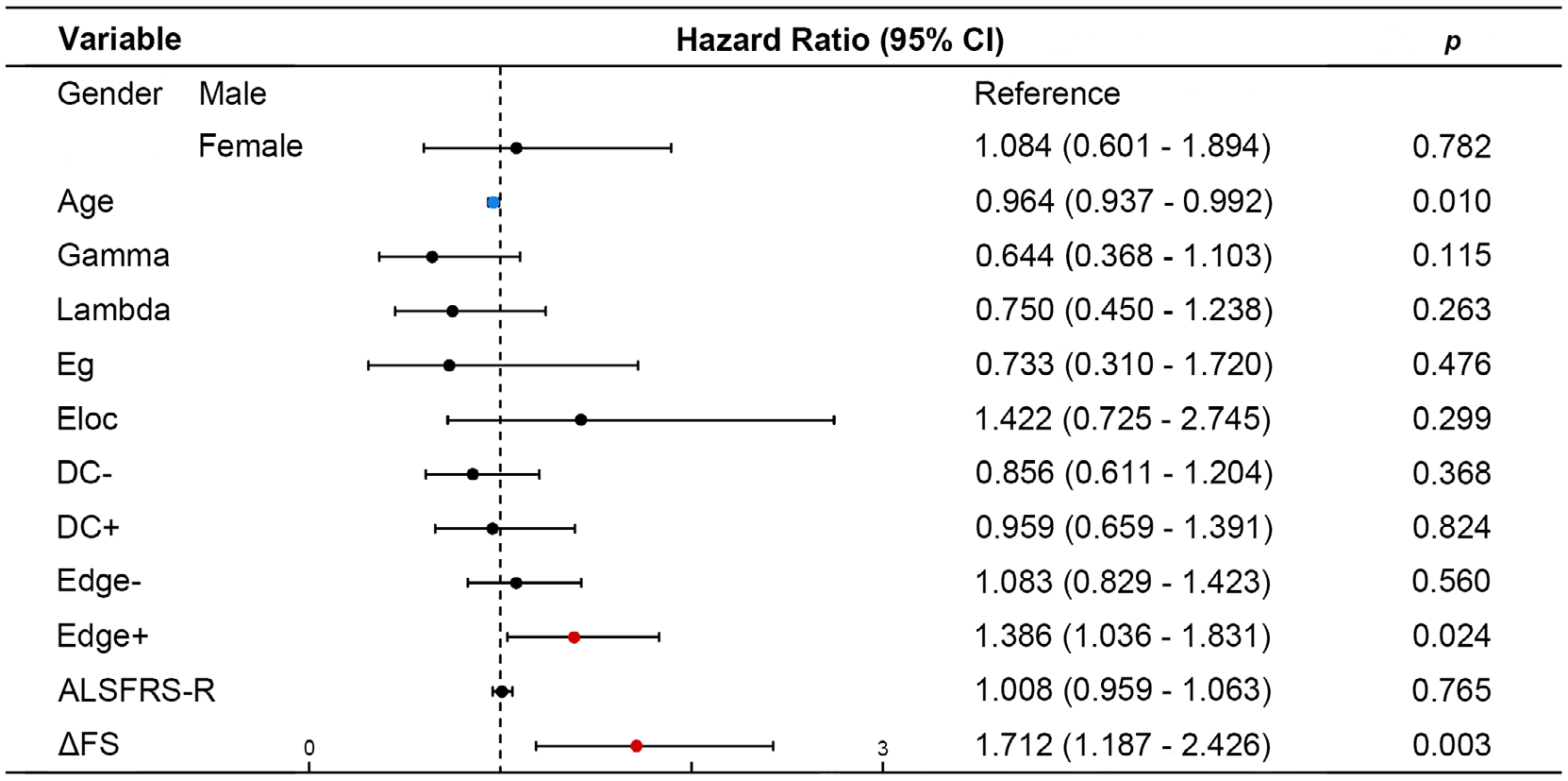
Final Cox proportional hazards model predicting risk of mortality in ALS patients, presented as its exponentiated coefficients (hazard ratios with 95% CI). CI, confidence interval; DC‐, average decrease in DC; DC+, average increase in DC; Edge−, average decrease in edge connectivity strength; Edge+, average increase in edge connectivity strength.

We identified three independent prognostic factors associated with overall survival in ALS patients: age, ΔFS, and average increase in edge connectivity strength (Figure 5). Specifically, a higher average increase in edge connectivity strength and a greater ΔFS score were linked to an increased risk of mortality, whereas older age was associated with a reduced risk of mortality.

## 4 Discussion

This study revealed disrupted topological organization in glucose metabolism networks among ALS patients, characterized by reduced small‐worldness (lower Sigma and Gamma, higher Lambda) and decreased global and local efficiency. These changes correlated with clinical severity, including ΔFS and ALSFRS‐R scores. Regionally, ALS patients showed both increased and decreased DC across the brain. Spatial correlation analyses linked DC alterations to GABAa and mGluR5 receptor expression, while transcriptional associations highlighted related immune, synaptic, and protein regulation pathways. Bidirectional changes in connectivity strength were observed, with increased average strength independently predicting poorer survival.

The glucose metabolism covariance networks of both groups displayed a small‐world architecture characterized by high Gamma and low Lambda, or alternatively, by a Sigma (i.e., Gamma/Lambda) greater than 1. This type of network organization facilitates both specialized (segregated) and integrated information processing, maximizing information propagation efficiency while minimizing wiring costs [18]. Despite a small‐world architecture, notable differences in small‐world parameters were observed between the two groups. Compared to HC, ALS patients showed increased Lambda, which aligns with the observed decrease in Eg. These results were consistent with previous structural and diffusion MRI‐based network analyses [19, 20, 21] and indicate reduced signal propagation speed and synchronizability in ALS. The observed increase in Lambda and decrease in Eg are likely attributable to a reduction of long‐range connections. Given the positive correlation between Eg and ALSFRS‐R score, it is reasonable to hypothesize that the Eg reduction may play a role in the motor impairments in ALS. Compared to HC, ALS patients also showed decreased Gamma, which aligns with the observed decrease in Eloc. These results are consistent with previous diffusion MRI‐based network analyses [19, 20, 21, 22] and indicate a reduction in local cliquishness, decreasing the robustness of the local network in the presence of node damage. These findings, however, contrast with our previous structural covariance network analysis, which demonstrated a significant increase in Gamma and Eloc [12]. The neural mechanisms underlying this discrepancy remain uncertain, but may relate to differences in clinical characteristics and methodology (e.g., gray matter volume vs. standardized uptake values, group‐based vs. individualized networks). These findings, along with the decrease in the Sigma, suggest that glucose metabolism covariance networks in ALS patients departed from an optimal small‐world configuration, disrupting both global integration and local segregation. Moreover, all global network parameters correlated significantly withΔFS scores, implying that changes in these parameters contribute to the worsening of motor impairments and could serve as potential biomarkers for monitoring disease progression.

Compared to HC, ALS patients showed decreased DC in multiple regions, including the right precentral gyrus, left middle frontal gyrus, bilateral postcentral gyrus, thalamus, and caudate. These findings align with hypometabolism and gray matter atrophy shown by previous studies [23, 24, 25, 26]. The precentral gyrus is critical for descending motor commands necessary for voluntary movement and is integral to various motor functions [27]. The middle frontal gyrus participates in the planning and preparatory stages of movement, executing complex movements and coordinating muscle groups [28]. Consequently, DC reductions in these regions may contribute to motor dysfunction, leading to symptoms such as limb paralysis. The postcentral gyrus exhibits significant connectivity with the precentral gyrus and plays a crucial role in sensorimotor integration [29]. Decreased DC in the postcentral gyrus could potentially contribute to abnormal sensorimotor integration in ALS. Our discovery of a negative correlation between DC in the left postcentral gyrus and ΔFS supports this hypothesis, suggesting that DC in this area might serve as an indicator of motor impairment progression due to deteriorating sensorimotor integration.

The gene set associated with DC reductions in ALS patients was enriched in several GO processes related to both intercellular and intracellular immune responses. One key process identified was monoatomic ion homeostasis, essential for cellular stability and biochemical reactions [30]. Fluctuations in calcium ions can trigger apoptosis‐related hydrolases, which degrade membrane and cytoskeletal proteins, inducing cell death [31]. This apoptotic process helps eliminate intracellular stressors that could disrupt homeostasis. However, impaired apoptosis can lead to stressor leakage, activating glial cells and intercellular immune signaling. Activated glial cells can then release immune signaling molecules, including neurotransmitters and pro‐inflammatory cytokines, involving processes such as “fatty acid metabolism” and “activation of immune response” [32, 33]. Subsequently, processes like the “transmembrane receptor protein tyrosine kinase signaling pathway” may recognize immune signaling molecules and activate intracellular tyrosine kinase activity, thereby modulating the activity of immune cells such as leukocytes. If cellular stressors are not effectively cleared by immune cells, persistent neuroinflammation or the invasion of these stressors into healthy neurons can lead to tissue damage. Overall, our findings suggest that a cascade of intercellular and intracellular immune responses is triggered by pathological processes, such as TDP‐43 accumulation, leading to localized neuroinflammation and neuronal injury, which manifests as reduced DC in ALS.

Compared to HC, ALS patients exhibited increased DC in some motor regions, such as the left putamen, right pars opercularis, and bilateral pars triangularis. These findings align with previous studies reporting increased activations in these regions [34, 35]. The putamen is critical for motor functions, particularly in coordinating movement initiation and execution, and integrating sensory information with motor planning [36]. The inferior frontal gyrus, particularly the pars opercularis and pars triangularis, participates in the planning and execution of speech movements [36]. The increased DC in these regions may reflect a compensatory response for mitigating progressive motor dysfunction. Meanwhile, ALS patients also showed increased DC in some key nodes of the default mode network (DMN), including the angular gyrus and medial superior frontal gyrus. These findings are consistent with previous studies reporting increased functional connectivity in the medial superior frontal gyrus [37]. The DMN is recognized for its pivotal role in various cognitive processes and psychological activities [38]. Increased DC in DMN regions may represent compensatory processes to counteract cognitive impairments in ALS. Additionally, we noted increased DC of the right superior occipital gyrus, which may serve as a compensatory mechanism to maintain normal visual processes supporting cognition. This interpretation is supported by the positive correlation between DC of this region and MMSE scores.

The gene set associated with increased DC in ALS patients was enriched in several GO processes related to trans‐synaptic signal transduction. These include processes such as “modulation of chemical synaptic transmission”, “trans‐synaptic signaling”, and “regulation of membrane potential”, all of which are integral to neural signal transmission across synapses. These processes may be linked to hyperactive synapses caused by elevated levels of excitatory neurotransmitter glutamate (or an imbalance in GABA and glutamate levels) [39]. This interpretation is supported by cross‐regional spatial correlation analyses, which revealed significant associations between the *t*‐values from between‐group contrast of DC and the expression of mGluR5 and GABAa receptors. Indeed, inefficient glutamate clearance prolongs synaptic presence, enhancing glutamatergic receptor activation and synaptic sensitivity. Persistent postsynaptic excitation likely underlies the increased DC in ALS. Although it initially compensates for functional deficits, this overactivation becomes neurotoxic due to sustained energy consumption and ionic imbalance, leading to oxidative stress, enzyme activation, and inflammation, progressively impairing synaptic function [40, 41]. Consequently, compensatory mechanisms may eventually become overwhelmed, leading to neuronal damage and degeneration. In addition, the set of genes is enriched in processes related to regulation of protein function, notably “regulation of establishment of protein localization”, which is relevant to the mislocalization and accumulation of TDP‐43. Protein phosphorylation, a key post‐translational modification, plays a critical role in regulating various protein functions, including activity, stability, localization, and aggregation propensity [42]. This may contribute to phosphorylated TDP‐43 aggregate formation, present in approximately 97% of ALS cases [43]. TDP‐43 pathologies may also impair glutamate clearance by interacting with proteins such as EAAT2, further exacerbating excitotoxic damage to neurons [44]. These interconnected processes of synaptic dysfunction and protein dysregulation likely contribute to increased DC (overactivation) and subsequent decrease in DC (neurodegeneration) in ALS.

This study revealed extensive changes in edge connectivity strength in ALS. Decreased connectivity strength was mainly found between the temporal pole and frontal cortices, consistent with a previous report of reduced functional connectivity between the orbitofrontal cortex and temporal pole in ALS‐FTD patients [45]. The frontal cortices and temporal pole play crucial roles in various cognitive functions, including executive functions, memory, and language [46, 47]. The decline in metabolic synchrony between the frontal cortices and temporal pole likely indicates a disruption in information transmission, contributing to the higher‐order cognitive dysfunctions. A significant decrease in connectivity strength was also observed between the limbic system and temporal lobe, involving the thalamus, anterior cingulate gyrus, middle cingulate gyrus, and temporal pole. These findings are consistent with previous studies showing cortical thickness in the anterior cingulate cortex [48]. The anterior cingulate gyrus, a key node of the limbic system, is involved in processing social information to guide self‐regulation and tracking feedback from others [49]. The middle cingulate gyrus is part of a distributed attentional network involved in the observation and execution of actions, while the temporal pole integrates sensory and emotional information to enhance social understanding and empathy [50, 51]. Decreased metabolic synchrony among these regions may contribute to deficits in emotional regulation, social cognition, and empathy in ALS. Meanwhile, significantly increased edge connectivity strength was primarily observed between the frontal lobe and several other brain regions, particularly the occipital lobe, basal ganglia, and limbic system. The neural substrates underlying this heightened metabolic synchrony remain unknown and may represent a kind of temporary compensatory mechanism arising from glutamate‐mediated excitotoxicity. This interpretation is supported by our Cox analysis result that an increase in connectivity strength was an independent risk factor for overall survival in these patients, with a greater increase in connectivity associated with lower overall survival. The potential interpretations of these results can be twofold. First, patients with faster progression may show greater compensation. Second, patients experiencing higher levels of glutamate‐mediated excitotoxicity tend to progress more rapidly, leading to a worse prognosis.

This study has several limitations. First, the cross‐sectional design limits our ability to characterize longitudinal changes in the topology of metabolism covariance networks in ALS patients; future longitudinal studies are needed to address this issue. Second, while DC maps were derived from our PET data, the neurotransmitter and gene expression maps were obtained from population‐level atlases. Hence, the spatial correlations did not account for individual variability, which may affect the interpretability of the findings.

In conclusion, this study reveals altered brain metabolic networks in ALS, linked to motor symptoms, disease progression, receptor expression, and mortality risk. Our findings may provide valuable biomarkers for monitoring ALS progression and suggest potential mechanistic pathways for developing innovative therapeutic strategies for this disorder.

## Ethics Statement

The study protocol received approval from the Ethics Committee and the Expert Committee at Xiangya Hospital, Central South University (IRB No. 202103191). Informed written consent was obtained from all participants.

## Consent

The authors have nothing to report.

## Conflicts of Interest

The authors declare no conflicts of interest.

## Supporting information


**Data S1:** cns70537‐sup‐0001‐DataS1.docx.

## Data Availability

The data that support the findings of the present study are available from the corresponding authors upon reasonable request.

## References

[1] M. C. Kiernan , S. Vucic , B. C. Cheah , et al., “Amyotrophic Lateral Sclerosis,” Lancet 377 (2011): 942–955. 21296405 10.1016/S0140-6736(10)61156-7

[2] B. Swinnen and W. Robberecht , “The Phenotypic Variability of Amyotrophic Lateral Sclerosis,” Nature Reviews Neurology 10 (2014): 661–670. 25311585 10.1038/nrneurol.2014.184

[3] A. Montuschi , B. Iazzolino , A. Calvo , et al., “Cognitive Correlates in Amyotrophic Lateral Sclerosis: A Population‐Based Study in Italy,” Journal of Neurology, Neurosurgery & Psychiatry 86 (2015): 168–173. 24769471 10.1136/jnnp-2013-307223

[4] L. H. Goldstein and S. Abrahams , “Changes in Cognition and Behaviour in Amyotrophic Lateral Sclerosis: Nature of Impairment and Implications for Assessment,” Lancet Neurology 12 (2013): 368–380. 23518330 10.1016/S1474-4422(13)70026-7

[5] T. Qiu , Y. Zhang , X. Tang , et al., “Precentral Degeneration and Cerebellar Compensation in Amyotrophic Lateral Sclerosis: A Multimodal MRI Analysis,” Human Brain Mapping 40 (2019): 3464–3474. 31020731 10.1002/hbm.24609 PMC6865414

[6] P. Bede , A. L. W. Bokde , S. Byrne , et al., “Multiparametric MRI Study of ALS Stratified for the *C9orf72* Genotype,” Neurology 81 (2013): 361–369. 23771489 10.1212/WNL.0b013e31829c5eee PMC3772833

[7] J. L. Chang , C. Lomen‐Hoerth , J. Murphy , et al., “A Voxel‐Based Morphometry Study of Patterns of Brain Atrophy in ALS and ALS/FTLD,” Neurology 65 (2005): 75–80. 16009889 10.1212/01.wnl.0000167602.38643.29

[8] F. Trojsi , F. Di Nardo , G. Caiazzo , et al., “Between‐Sex Variability of Resting State Functional Brain Networks in Amyotrophic Lateral Sclerosis (ALS),” Journal of Neural Transmission 128 (2021): 1881–1897. 34471976 10.1007/s00702-021-02413-0 PMC8571222

[9] A. Canosa , A. Martino , A. Giuliani , et al., “Brain Metabolic Differences Between Pure Bulbar and Pure Spinal ALS: A 2‐[18F]FDG‐PET Study,” Journal of Neurology 270 (2023): 953–959. 36322237 10.1007/s00415-022-11445-9 PMC9886651

[10] A. Sala , L. Iaccarino , P. Fania , et al., “Testing the Diagnostic Accuracy of [18F]FDG‐PET in Discriminating Spinal‐ and Bulbar‐Onset Amyotrophic Lateral Sclerosis,” European Journal of Nuclear Medicine and Molecular Imaging 46 (2019): 1117–1131. 30617963 10.1007/s00259-018-4246-2

[11] A. Canosa , A. Calvo , C. Moglia , et al., “Amyotrophic Lateral Sclerosis With SOD1 Mutations Shows Distinct Brain Metabolic Changes,” European Journal of Nuclear Medicine and Molecular Imaging 49 (2022): 2242–2250. 35076740 10.1007/s00259-021-05668-7 PMC9165265

[12] Y. Zhang , T. Qiu , X. Yuan , et al., “Abnormal Topological Organization of Structural Covariance Networks in Amyotrophic Lateral Sclerosis,” NeuroImage: Clinical 21 (2018): 101619. 30528369 10.1016/j.nicl.2018.101619 PMC6411656

[13] Z. Sha , D. Van Rooij , E. Anagnostou , et al., “Subtly Altered Topological Asymmetry of Brain Structural Covariance Networks in Autism Spectrum Disorder Across 43 Datasets From the ENIGMA Consortium,” Molecular Psychiatry 27 (2022): 2114–2125. 35136228 10.1038/s41380-022-01452-7 PMC9126820

[14] Y. Tang , H. Zhu , L. Xiao , et al., “Individual Cerebellar Metabolic Connectome in Patients With MTLE and NTLE Associated With Surgical Prognosis,” European Journal of Nuclear Medicine and Molecular Imaging 51 (2024): 3600–3616. 38805089 10.1007/s00259-024-06762-2

[15] L. Palaniyappan , B. Park , V. Balain , R. Dangi , and P. Liddle , “Abnormalities in Structural Covariance of Cortical Gyrification in Schizophrenia,” Brain Structure & Function 220 (2015): 2059–2071. 24771247 10.1007/s00429-014-0772-2 PMC4481329

[16] N. Tzourio‐Mazoyer , B. Landeau , D. Papathanassiou , et al., “Automated Anatomical Labeling of Activations in SPM Using a Macroscopic Anatomical Parcellation of the MNI MRI Single‐Subject Brain,” NeuroImage 15 (2002): 273–289. 11771995 10.1006/nimg.2001.0978

[17] A. Ludolph , V. Drory , O. Hardiman , et al., “A Revision of the El Escorial Criteria–2015,” Amyotrophic Lateral Sclerosis and Frontotemporal Degeneration 16 (2015): 291–292. 26121170 10.3109/21678421.2015.1049183

[18] S. Achard and E. Bullmore , “Efficiency and Cost of Economical Brain Functional Networks,” PLoS Computational Biology 3 (2007): e17. 17274684 10.1371/journal.pcbi.0030017 PMC1794324

[19] D. Dimond , A. Ishaque , S. Chenji , et al., “White Matter Structural Network Abnormalities Underlie Executive Dysfunction in Amyotrophic Lateral Sclerosis,” Human Brain Mapping 38 (2017): 1249–1268. 27796080 10.1002/hbm.23452 PMC6866969

[20] S. Basaia , F. Agosta , C. Cividini , et al., “Structural and Functional Brain Connectome in Motor Neuron Diseases: A Multicenter MRI Study,” Neurology 95 (2020): e2552–e2564. 32913015 10.1212/WNL.0000000000010731 PMC7682834

[21] F. Feng , G. Feng , J. Liu , et al., “Different Patterns of Structural Network Impairments in Two Amyotrophic Lateral Sclerosis Subtypes Driven by 18F‐Fluorodeoxyglucose Positron Emission Tomography/Magnetic Resonance Hybrid Imaging,” Brain Communications 6 (2024): fcae222. 39229489 10.1093/braincomms/fcae222 PMC11368155

[22] W. Li , Q. Wei , Y. Hou , et al., “Disruption of the White Matter Structural Network and Its Correlation With Baseline Progression Rate in Patients With Sporadic Amyotrophic Lateral Sclerosis,” Translational Neurodegeneration 10 (2021): 35. 34511130 10.1186/s40035-021-00255-0 PMC8436442

[23] J. A. Matías‐Guiu , V. Pytel , M. N. Cabrera‐Martín , et al., “Amyloid‐ and FDG‐PET Imaging in Amyotrophic Lateral Sclerosis,” European Journal of Nuclear Medicine and Molecular Imaging 43 (2016): 2050–2060. 27262702 10.1007/s00259-016-3434-1

[24] P. Bede , M. Elamin , S. Byrne , et al., “Basal Ganglia Involvement in Amyotrophic Lateral Sclerosis,” Neurology 81 (2013): 2107–2115. 24212388 10.1212/01.wnl.0000437313.80913.2c

[25] J. Brettschneider , D. J. Libon , J. B. Toledo , et al., “Microglial Activation and TDP‐43 Pathology Correlate With Executive Dysfunction in Amyotrophic Lateral Sclerosis,” Acta Neuropathologica 123 (2012): 395–407. 22210083 10.1007/s00401-011-0932-x PMC3595560

[26] K. Van Laere , A. Vanhee , J. Verschueren , et al., “Value of^18^ Fluorodeoxyglucose–Positron‐Emission Tomography in Amyotrophic Lateral Sclerosis: A Prospective Study,” JAMA Neurology 71 (2014): 553. 24615479 10.1001/jamaneurol.2014.62

[27] R. N. Lemon , “Descending Pathways in Motor Control,” Annual Review of Neuroscience 31 (2008): 195–218. 10.1146/annurev.neuro.31.060407.125547 18558853

[28] L. D. Pettit , M. E. Bastin , C. Smith , T. H. Bak , T. H. Gillingwater , and S. Abrahams , “Executive Deficits, Not Processing Speed Relates to Abnormalities in Distinct Prefrontal Tracts in Amyotrophic Lateral Sclerosis,” Brain 136 (2013): 3290–3304. 24056536 10.1093/brain/awt243

[29] M. Vukelić , R. Bauer , G. Naros , I. Naros , C. Braun , and A. Gharabaghi , “Lateralized Alpha‐Band Cortical Networks Regulate Volitional Modulation of Beta‐Band Sensorimotor Oscillations,” NeuroImage 87 (2014): 147–153. 24121086 10.1016/j.neuroimage.2013.10.003

[30] N. Nelson , “Metal Ion Transporters and Homeostasis,” EMBO Journal 18 (1999): 4361–4371. 10449402 10.1093/emboj/18.16.4361 PMC1171511

[31] D. E. Clapham , “Calcium Signaling,” Cell 131 (2007): 1047–1058. 18083096 10.1016/j.cell.2007.11.028

[32] Y. Dong and E. N. Benveniste , “Immune Function of Astrocytes,” Glia 36 (2001): 180–190. 11596126 10.1002/glia.1107

[33] G. W. Kreutzberg , “Microglia: A Sensor for Pathological Events in the CNS,” Trends in Neurosciences 19 (1996): 312–318. 8843599 10.1016/0166-2236(96)10049-7

[34] B. Mohammadi , K. Kollewe , D. M. Cole , et al., “Amyotrophic Lateral Sclerosis Affects Cortical and Subcortical Activity Underlying Motor Inhibition and Action Monitoring: ALS and Motor Inhibition,” Human Brain Mapping 36 (2015): 2878–2889. 25913637 10.1002/hbm.22814 PMC6869134

[35] H. Li , Y. Chen , Y. Li , et al., “Altered Cortical Activation During Action Observation in Amyotrophic Lateral Sclerosis Patients: A Parametric Functional MRI Study,” European Radiology 25 (2015): 2584–2592. 25875284 10.1007/s00330-015-3671-x

[36] C. J. Price , “The Anatomy of Language: A Review of 100 fMRI Studies Published in 2009,” Annals of the New York Academy of Sciences 1191 (2010): 62–88. 20392276 10.1111/j.1749-6632.2010.05444.x

[37] V. Castelnovo , E. Canu , D. Calderaro , et al., “Progression of Brain Functional Connectivity and Frontal Cognitive Dysfunction in ALS,” NeuroImage: Clinical 28 (2020): 102509. 33395998 10.1016/j.nicl.2020.102509 PMC7708866

[38] V. Menon , “The Triple Network Model, Insight, and Large‐Scale Brain Organization in Autism,” Biological Psychiatry 84 (2018): 236–238. 30071947 10.1016/j.biopsych.2018.06.012 PMC6345251

[39] L. Cheng , Y. Yuan , X. Tang , et al., “Structural and Functional Underpinnings of Precentral Abnormalities in Amyotrophic Lateral Sclerosis,” European Journal of Neurology 28 (2021): 1528–1536. 33404153 10.1111/ene.14717

[40] J. T. Coyle and P. Puttfarcken , “Oxidative Stress, Glutamate, and Neurodegenerative Disorders,” Science 262 (1993): 689–695. 7901908 10.1126/science.7901908

[41] P. Van Cutsem , M. Dewil , W. Robberecht , and L. Bosch , “Excitotoxicity and Amyotrophic Lateral Sclerosis,” Neurodegenerative Diseases 2 (2005): 147–159. 16909020 10.1159/000089620

[42] E. Buratti , “TDP‐43 Post‐Translational Modifications in Health and Disease,” Expert Opinion on Therapeutic Targets 22 (2018): 279–293. 29431050 10.1080/14728222.2018.1439923

[43] O. J. Ziff , J. Neeves , J. Mitchell , et al., “Integrated Transcriptome Landscape of ALS Identifies Genome Instability Linked to TDP‐43 Pathology,” Nature Communications 14 (2023): 2176. 10.1038/s41467-023-37630-6 PMC10119258 37080969

[44] D. C. Diaper , Y. Adachi , L. Lazarou , et al., “Drosophila TDP‐43 Dysfunction in Glia and Muscle Cells Cause Cytological and Behavioural Phenotypes That Characterize ALS and FTLD,” Human Molecular Genetics 22 (2013): 3883–3893. 23727833 10.1093/hmg/ddt243 PMC3766182

[45] R. F. Smallwood Shoukry , M. G. Clark , and M. K. Floeter , “Resting State Functional Connectivity Is Decreased Globally Across the C9orf72 Mutation Spectrum,” Frontiers in Neurology 11 (2020): 598474. 33329355 10.3389/fneur.2020.598474 PMC7710968

[46] A. L. Jouen , T. M. Ellmore , C. J. Madden , C. Pallier , P. F. Dominey , and J. Ventre‐Dominey , “Beyond the Word and Image: Characteristics of a Common Meaning System for Language and Vision Revealed by Functional and Structural Imaging,” NeuroImage 106 (2015): 72–85. 25463475 10.1016/j.neuroimage.2014.11.024

[47] Y. Yin , Z. Hou , X. Wang , Y. Sui , and Y. Yuan , “The BDNF Val66Met Polymorphism, Resting‐State Hippocampal Functional Connectivity and Cognitive Deficits in Acute Late‐Onset Depression,” Journal of Affective Disorders 183 (2015): 22–30. 26000753 10.1016/j.jad.2015.04.050

[48] K. Placek , M. Benatar , J. Wuu , et al., “Machine Learning Suggests Polygenic Risk for Cognitive Dysfunction in Amyotrophic Lateral Sclerosis,” EMBO Molecular Medicine 13 (2021): e12595. 33270986 10.15252/emmm.202012595 PMC7799365

[49] M. A. J. Apps , M. F. S. Rushworth , and S. W. C. Chang , “The Anterior Cingulate Gyrus and Social Cognition: Tracking the Motivation of Others,” Neuron 90 (2016): 692–707. 27196973 10.1016/j.neuron.2016.04.018 PMC4885021

[50] G. Di Cesare , M. Marchi , G. Lombardi , M. Gerbella , A. Sciutti , and G. Rizzolatti , “The Middle Cingulate Cortex and Dorso‐Central Insula: A Mirror Circuit Encoding Observation and Execution of Vitality Forms,” Proceedings of the National Academy of Sciences of the United States of America 118 (2021): e2111358118. 34716272 10.1073/pnas.2111358118 PMC8612212

[51] K. P. Rankin , M. L. Gorno‐Tempini , S. C. Allison , et al., “Structural Anatomy of Empathy in Neurodegenerative Disease,” Brain 129 (2006): 2945–2956. 17008334 10.1093/brain/awl254 PMC2562652

