# Supplementary material for: Disrupted Glucose Metabolism Covariance Network in Amyotrophic Lateral Sclerosis
Source: CNS Neurosci Ther. 2025 Jul 28;31(7):e70537. doi: 10.1111/cns.70537 (PMC12304423; doi:10.1111/cns.70537)
Supplement: Supplementary file 1 — Data S1: cns70537‐sup‐0001‐DataS1.docx. [file CNS-31-e70537-s001.docx]

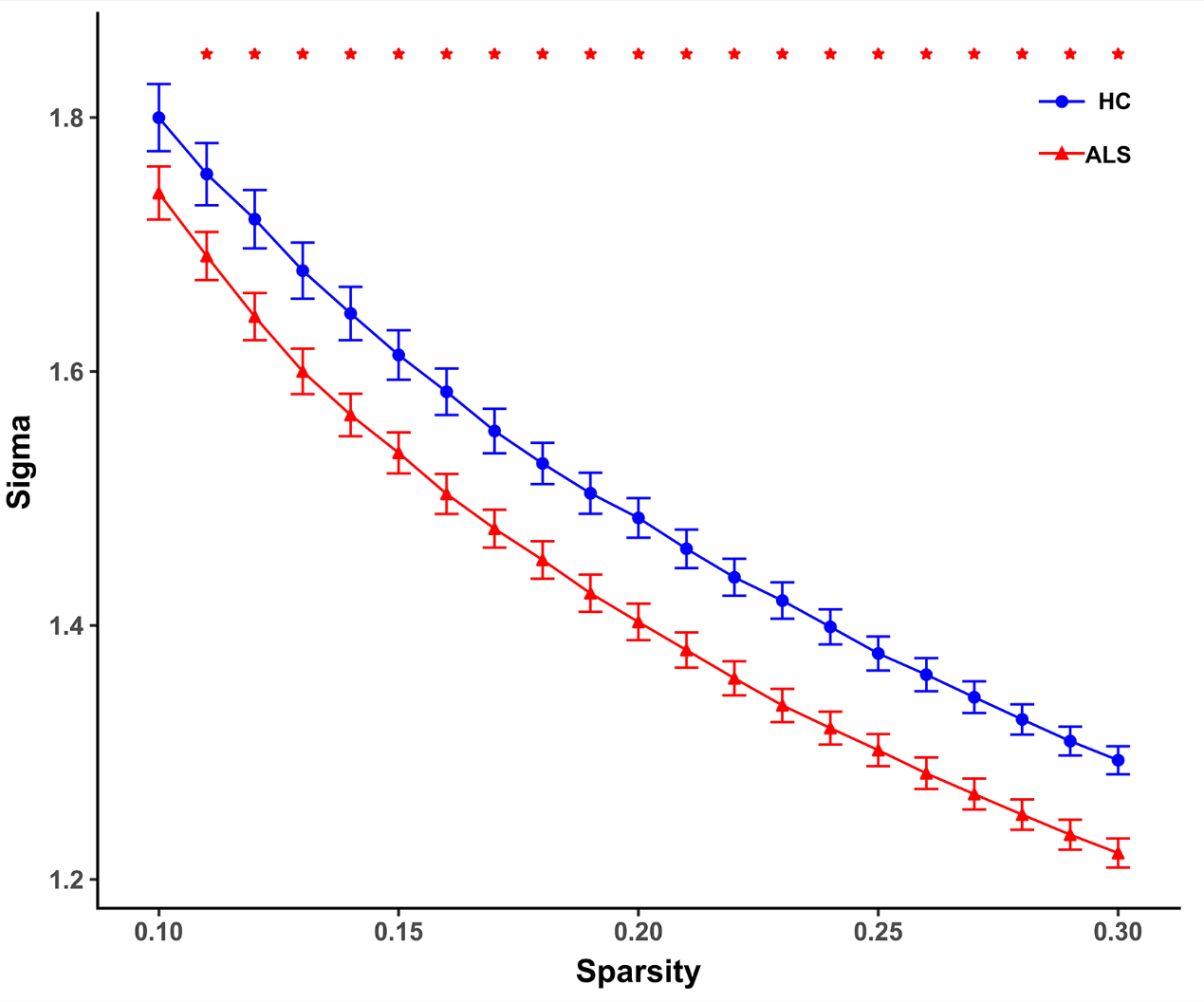


**Figure S1.** Differences in Sigma (i.e., small-world index) between patients with ALS and HC. Patients with ALS showed significantly lower Sigma across several sparsity levels.


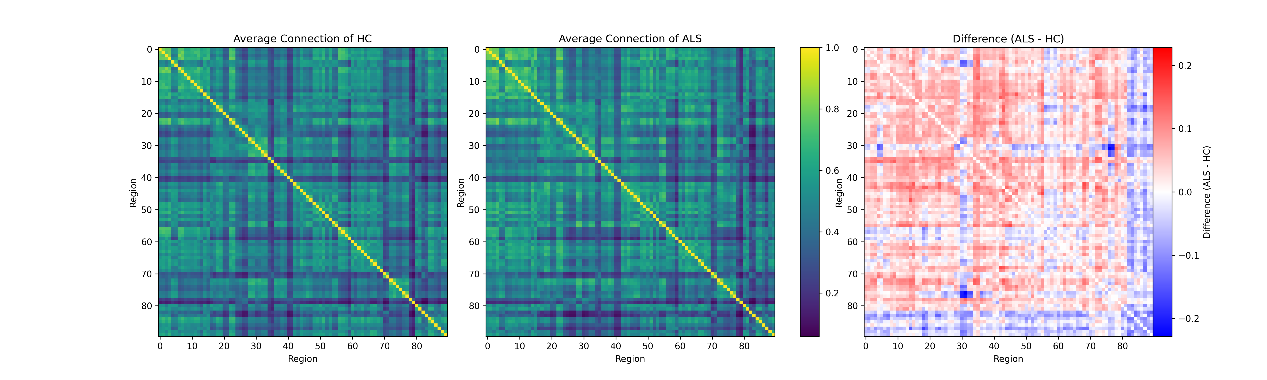


**Figure S2.** Group-level comparison of average connectivity matrices. The left and middle panels show the mean connectivity matrices for HC and patients with ALS, respectively. Each element reflects the average connection strength between two brain regions across individuals in each group. The right panel displays the difference matrix (ALS-HC), highlighting the regional alterations in connectivity strength.

**Table S1.** Abbreviations for brain regions of the automated anatomical labelling (AAL) atlas

| **Abbreviation** | **Region** |
| --- | --- |
| PreCG | Precentral gyrus |
| SFGdor | Superior frontal gyrus, dorsolateral |
| ORBsup | Superior frontal gyrus, orbital part |
| MFG | Middle frontal gyrus |
| ORBmid | Middle frontal gyrus, orbital part |
| IFGoperc | Inferior frontal gyrus, opercular part |
| IFGtriang | Inferior frontal gyrus, triangular part |
| ORBinf | Inferior frontal gyrus, orbital part |
| ROL | Rolandic operculum |
| SMA | Supplementary motor area |
| OLF | Olfactory cortex |
| SFGmed | Superior frontal gyrus, medial |
| ORBsupmed | Superior frontal gyrus, medial orbital |
| REC | Gyrus rectus |
| INS | Insula |
| ACG | Anterior cingulate & paracingulate gyri |
| DCG | Middle cingulate & paracingulate gyri |
| PCG | Posterior cingulate gyrus |
| HIP | Hippocampus |
| PHG | Parahippocampal gyrus |
| AMYG | Amygdala |
| CAL | Calcarine fissure and surrounding cortex |
| CUN | Cuneus |
| LING | Lingual gyrus |
| SOG | Superior occipital gyrus |
| MOG | Middle occipital gyrus |
| IOG | Inferior occipital gyrus |
| FFG | Fusiform gyrus |
| PoCG | Postcentral gyrus |
| SPG | Superior parietal gyrus |
| IPL | Inferior parietal gyrus, excluding supramarginal and angular gyri |
| SMG | SupraMarginal gyrus |
| ANG | Angular gyrus |
| PCUN | Precuneus |
| PCL | Paracentral lobule |
| CAU | Caudate nucleus |
| PUT | Lenticular nucleus, Putamen |
| PAL | Lenticular nucleus, Pallidum |
| THA | Thalamus |
| HES | Heschl’s gyrus |
| STG | Superior temporal gyrus |
| TPOsup | Temporal pole: superior temporal gyrus |
| MTG | Middle temporal gyrus |
| TPOmid | Temporal pole: middle temporal gyrus |
| ITG | Inferior temporal gyrus |

**Table S2.** Differences in nodal degree centrality between patients with ALS and HC

| **Region** | **T** | | ***p*-value (FDR)** | **Δ** |
| --- | --- | --- | --- | --- |
| PreCG.R | 2.4109 | | 0.0499 | -0.4012 |
| MFG.L | 4.1921 | | 0.0003 | -0.5430 |
| MFG.R | 2.2186 | | 0.0705 | -0.3366 |
| ACG.L | 7.4436 | | <0.0001 | -1.1310 |
| ACG.R | 6.4323 | | <0.0001 | -1.0512 |
| DCG.L | 2.3667 | | 0.0536 | -0.4084 |
| DCG.R | 4.5927 | | 0.0001 | -0.6940 |
| PoCG.L | 2.4909 | | 0.0463 | -0.6274 |
| PoCG.R | 2.4394 | | 0.0478 | -0.6342 |
| SPG.R | 3.0360 | | 0.0113 | -0.3718 |
| CAU.L | 4.1768 | | 0.0003 | -0.2056 |
| CAU.R | 3.7971 | | 0.0010 | -0.2897 |
| THA.L | 6.4790 | | <0.0001 | -1.0267 |
| THA.R | 4.9194 | | <0.0001 | -0.7909 |
| TPOsup.L | 3.2738 | | 0.0060 | -0.6131 |
| TPOsup.R | 4.8420 | | <0.0001 | -0.7903 |
| TPOmid.R | 4.6379 | | 0.0001 | -0.8352 |
| ORBmid.L | -4.4843 | | 0.0001 | 0.7834 |
| IFGoperc.R | -3.9210 | | 0.0007 | 0.7657 |
| IFGtriang.L | -2.6495 | | 0.0308 | 0.3993 |
| IFGtriang.R | -2.7040 | | 0.0299 | 0.3833 |
| ORBinf.L | | -2.1876 | 0.0740 | 0.5289 |
| ORBinf.R | | -5.7001 | <0.0001 | 1.1738 |
| OLF.L | | -2.2467 | 0.0676 | 0.2454 |
| SFGmed.L | | -2.0413 | 0.0975 | 0.3787 |
| SFGmed.R | | -3.6565 | 0.0016 | 0.5324 |
| REC.R | | -2.0724 | 0.0929 | 0.2691 |
| HIP.R | | -2.1584 | 0.0774 | 0.3735 |
| PHG.L | | -2.3598 | 0.0536 | 0.4279 |
| PHG.R | | -2.0066 | 0.1032 | 0.3774 |

*Note:* Between-group differences were examined using two-sample t-test. The results were corrected for multiple comparisons using FDR. L, left; R, right; T, t-statistic; Δ, difference in average values between ALS patients and HC.

**Table S3.** Altered edge connectivity strength in patients with ALS compared to HC

| **Lobe** | **Edge connectivity** | | **Direction** |
| --- | --- | --- | --- |
| **Frontal Lobe** | ORBinf.L | ORBsup.L | increase |
|  | ORBinf.R | MFG.R | increase |
|  | ORBinf.R | ORBmid.L | increase |
|  | ORBinf.R | ORBmid.R | increase |
|  | ORBinf.R | ORBsup.L | increase |
|  | ORBinf.R | ORBsup.R | increase |
|  | ORBinf.R | PreCG.R | increase |
|  | ORBinf.R | SFGdor.L | increase |
|  | ORBinf.R | SFGdor.R | increase |
|  | ORBmid.L | SFGdor.L | increase |
|  | ORBmid.L | SFGdor.R | increase |
|  | ORBmid.R | SFGdor.L | increase |
|  | ORBmid.R | SFGdor.R | increase |
|  | SFGmed.L | ORBinf.R | increase |
|  | SFGmed.R | ORBinf.R | increase |
| **Basal ganglia** | PAL.L | ACG.L | decrease |
|  | PAL.L | ACG.R | decrease |
|  | CAU.R | ORBmid.R | increase |
|  | CAU.R | ORBsup.L | increase |
|  | PAL.L | CUN.L | increase |
|  | PAL.L | CUN.R | increase |
|  | PAL.L | LING.R | increase |
|  | PAL.L | ORBinf.L | increase |
|  | PAL.R | CUN.L | increase |
|  | PUT.L | CAL.R | increase |
|  | PUT.L | CUN.L | increase |
|  | PUT.L | IFGoperc.R | increase |
|  | PUT.L | IFGtriang.L | increase |
|  | PUT.L | IFGtriang.R | increase |
|  | PUT.L | ORBinf.L | increase |
|  | PUT.L | ORBinf.R | increase |
|  | PUT.L | PCG.L | increase |
|  | PUT.L | PCG.R | increase |
|  | PUT.L | PCL.L | increase |
|  | PUT.L | SFGdor.L | increase |
|  | PUT.L | SFGdor.R | increase |
|  | PUT.L | SFGmed.R | increase |
|  | PUT.L | SMA.L | increase |
|  | PUT.L | SMA.R | increase |
|  | PUT.L | SMG.L | increase |
|  | PUT.R | CAL.R | increase |
|  | PUT.R | CUN.L | increase |
|  | PUT.R | IFGoperc.R | increase |
|  | PUT.R | IFGtriang.L | increase |
|  | PUT.R | IFGtriang.R | increase |
|  | PUT.R | ORBinf.L | increase |
|  | PUT.R | ORBinf.R | increase |
|  | PUT.R | ORBmid.L | increase |
|  | PUT.R | PCG.L | increase |
|  | PUT.R | PCG.R | increase |
|  | PUT.R | PCL.L | increase |
|  | PUT.R | PCL.R | increase |
|  | PUT.R | SFGdor.R | increase |
|  | PUT.R | SFGmed.R | increase |
|  | PUT.R | SMA.L | increase |
|  | PUT.R | SMA.R | increase |
|  | PUT.R | SMG.L | increase |
| **Limbic system** | ACG.L | ORBsup.L | decrease |
|  | ACG.R | ORBsup.L | decrease |
|  | THA.L | ACG.L | decrease |
|  | THA.L | ACG.R | decrease |
|  | THA.L | DCG.L | decrease |
|  | THA.L | DCG.R | decrease |
|  | THA.L | HIP.L | decrease |
|  | THA.R | ACG.L | decrease |
|  | THA.R | ACG.R | decrease |
|  | THA.R | DCG.L | decrease |
|  | THA.R | DCG.R | decrease |
|  | ACG.R | REC.R | increase |
|  | HIP.R | SFGmed.R | increase |
|  | PCG.L | ORBmid.R | increase |
|  | PCG.L | REC.L | increase |
|  | PCG.L | SMA.L | increase |
|  | PCG.R | ORBinf.L | increase |
|  | PCG.R | ORBinf.R | increase |
|  | PCG.R | ORBmid.L | increase |
|  | PCG.R | ORBmid.R | increase |
|  | PCG.R | ORBsup.L | increase |
|  | PCG.R | ORBsup.R | increase |
|  | PCG.R | SFGdor.L | increase |
|  | PCG.R | SFGdor.R | increase |
|  | PCG.R | SFGmed.R | increase |
|  | PHG.L | SFGmed.R | increase |
| **Occipital Lobe** | CAL.L | IFGoperc.R | increase |
|  | CAL.L | ORBinf.R | increase |
|  | CAL.L | ORBmid.L | increase |
|  | CAL.L | PCG.L | increase |
|  | CAL.L | SFGdor.R | increase |
|  | CAL.L | SFGmed.R | increase |
|  | CAL.R | IFGoperc.L | increase |
|  | CAL.R | IFGoperc.R | increase |
|  | CAL.R | IFGtriang.L | increase |
|  | CAL.R | IFGtriang.R | increase |
|  | CAL.R | OLF.R | increase |
|  | CAL.R | ORBinf.L | increase |
|  | CAL.R | ORBinf.R | increase |
|  | CAL.R | ORBmid.L | increase |
|  | CAL.R | PCG.L | increase |
|  | CAL.R | PCG.R | increase |
|  | CAL.R | ROL.R | increase |
|  | CAL.R | SFGdor.R | increase |
|  | CAL.R | SFGmed.L | increase |
|  | CAL.R | SFGmed.R | increase |
|  | CAL.R | SMA.L | increase |
|  | CAL.R | SMA.R | increase |
|  | CUN.L | CAL.R | increase |
|  | CUN.L | ORBinf.L | increase |
|  | CUN.L | PCG.R | increase |
|  | CUN.L | SFGmed.R | increase |
|  | CUN.R | INS.L | increase |
|  | CUN.R | PHG.R | increase |
|  | FFG.L | CUN.R | increase |
|  | FFG.L | IFGtriang.R | increase |
|  | FFG.R | CAL.R | increase |
|  | FFG.R | CUN.L | increase |
|  | FFG.R | CUN.R | increase |
|  | FFG.R | IFGoperc.L | increase |
|  | FFG.R | IFGoperc.R | increase |
|  | FFG.R | IFGtriang.L | increase |
|  | FFG.R | IFGtriang.R | increase |
|  | FFG.R | INS.R | increase |
|  | FFG.R | ORBinf.L | increase |
|  | FFG.R | PCG.L | increase |
|  | FFG.R | PCG.R | increase |
|  | FFG.R | SFGmed.R | increase |
|  | FFG.R | SMA.R | increase |
|  | LING.R | CUN.L | increase |
|  | LING.R | ORBinf.L | increase |
| **Parietal Lobe** | PoCG.L | SMA.L | decrease |
|  | SPG.R | SMA.L | decrease |
|  | ANG.L | ORBinf.R | increase |
|  | PCL.L | CAL.L | increase |
|  | PCL.L | CAL.R | increase |
|  | PCL.L | FFG.R | increase |
|  | PCL.L | HIP.R | increase |
|  | PCL.L | OLF.R | increase |
|  | PCL.L | PCG.R | increase |
|  | PCL.R | CAL.L | increase |
|  | PCL.R | PCG.L | increase |
|  | PCL.R | PCG.R | increase |
|  | SMG.L | CAL.R | increase |
|  | SMG.L | CUN.L | increase |
|  | SMG.L | FFG.R | increase |
|  | SMG.L | HIP.L | increase |
|  | SMG.L | HIP.R | increase |
|  | SMG.L | OLF.R | increase |
|  | SMG.L | ORBinf.L | increase |
|  | SMG.L | PCG.R | increase |
|  | SMG.L | SFGmed.R | increase |
| **Temporal Lobe** | ITG.L | ACG.L | decrease |
|  | ITG.L | ACG.R | decrease |
|  | ITG.L | TPOmid.R | decrease |
|  | ITG.L | TPOsup.R | decrease |
|  | ITG.R | ACG.L | decrease |
|  | MTG.L | ACG.L | decrease |
|  | MTG.L | TPOsup.L | decrease |
|  | MTG.L | TPOsup.R | decrease |
|  | TPOmid.R | ACG.L | decrease |
|  | TPOmid.R | ACG.R | decrease |
|  | TPOmid.R | CUN.L | decrease |
|  | TPOmid.R | CUN.R | decrease |
|  | TPOmid.R | DCG.R | decrease |
|  | TPOmid.R | ORBinf.L | decrease |
|  | TPOmid.R | ORBsupmed.R | decrease |
|  | TPOmid.R | PCL.L | decrease |
|  | TPOmid.R | PCL.R | decrease |
|  | TPOmid.R | PCUN.L | decrease |
|  | TPOmid.R | PCUN.R | decrease |
|  | TPOmid.R | SFGmed.R | decrease |
|  | TPOmid.R | SMA.L | decrease |
|  | TPOmid.R | SMA.R | decrease |
|  | TPOmid.R | SMG.L | decrease |
|  | TPOmid.R | STG.L | decrease |
|  | TPOsup.L | ACG.L | decrease |
|  | TPOsup.L | ACG.R | decrease |
|  | TPOsup.L | DCG.L | decrease |
|  | TPOsup.L | DCG.R | decrease |
|  | TPOsup.L | HIP.L | decrease |
|  | TPOsup.R | ACG.L | decrease |
|  | TPOsup.R | ACG.R | decrease |
|  | TPOsup.R | CUN.L | decrease |
|  | TPOsup.R | CUN.R | decrease |
|  | TPOsup.R | DCG.L | decrease |
|  | TPOsup.R | DCG.R | decrease |
|  | TPOsup.R | HIP.L | decrease |
|  | TPOsup.R | IPL.L | decrease |
|  | TPOsup.R | MFG.R | decrease |
|  | TPOsup.R | ORBinf.L | decrease |
|  | TPOsup.R | ORBsupmed.R | decrease |
|  | TPOsup.R | PCL.L | decrease |
|  | TPOsup.R | PCUN.L | decrease |
|  | TPOsup.R | PCUN.R | decrease |
|  | TPOsup.R | SFGmed.L | decrease |
|  | TPOsup.R | SFGmed.R | decrease |
|  | TPOsup.R | SMA.L | decrease |
|  | TPOsup.R | SMA.R | decrease |
|  | TPOsup.R | SMG.L | decrease |
|  | TPOsup.R | STG.L | decrease |
|  | HES.L | ACG.R | increase |
|  | STG.L | PCG.R | increase |
|  | STG.R | PCG.R | increase |

*Note*: L, left; R, right.
